# Elucidation of neutrophils-mediated effect of MMP-2 on lung epithelial cells; implications for acute respiratory distress syndrome and severe dengue pathogenesis

**DOI:** 10.3389/fcimb.2025.1670259

**Published:** 2025-12-11

**Authors:** Rituraj Niranjan, Khashpatika Ganesh, Anupama Sunil, Vyshali Murugasamy, Pitchavel Vidhyapriya, Muthukumaravel Subramaniam, Ashwani Kumar

**Affiliations:** 1 Immunology Laboratories, Division of Microbiology and Immunology, ICMR-Vector Control Research Centre, Puducherry, India; 2 Division of Immunology, Indian Council of Medical Research (ICMR)-National Institute of Research in Tribal Health, Jabalpur, India; 3 Division of Molecular Biology and Immunology, ICMR- Vector Control Research Centre (ICMR-VCRC), Puducherry, India; 4 Division of Molecular Epidemiology, Indian Council of Medical Research (ICMR)-Vector Control Research Centre, Puducherry, India

**Keywords:** neutrophils, dengue pathogenesis, MMP-2, acute respiratory distress syndrome, lung epithelial cells, Atorvastatin, NS-1 Antigen, HL-60

## Abstract

Dengue is a vector-borne viral fever that is spread by the bites of Aedes mosquitoes. Matrix metalloproteinases (MMPs) play a significant role in the beginning and development of dengue pathogenesis, but their precise functions with respect to neutrophil-associated lung pathology are not understood. In the present study, we, for the first time, report that *in-vitro* cultured neutrophils secrete MMP-2 in response to dengue virus NS1 antigens in culture medium (the secretome). We have assessed the effect of neutrophil secretome on the apoptosis of A549 lung epithelial cells and found that it causes their death. This suggested that neutrophils cause apoptosis of lung epithelial cells via MMP-2-mediated mechanisms. The exposure of purified MMP-2 protein has also caused cell death of A549 epithelial cells by increasing the mRNA expression pattern of apoptotic genes. Atorvastatin (10 µM) treatment attenuated the change in the release of MMP-2 and further decreased the apoptosis of lung epithelial cells. Interestingly, it was seen that MMP-14 alone and NS1 antigen alone do not cause the apoptosis of lung epithelial cells, ruling out their direct involvement. The interactions of CD31-positive neutrophils in lungs and MMP-2 expression were also found in NS1-injected mice, supporting their role in *in-vivo* situations. In conclusion, this study suggests that the interaction of NS1-activated neutrophils with the alveolar epithelial cells participates in the lung pathogenesis involved in the acute respiratory distress syndrome (ARDS) in severe dengue disease. The present results are encouraging; however, further investigations are required to clarify the findings.

## 1 Introduction

The genus *Flavivirus* of the *Flaviviridae* family embraces close to 74 arboviruses with their enzootic cycles in various arthropods, including tick–borne, mosquito-borne, and several with unknown arthropod hosts (Alghsham et al., 2023; Tejashree et al., 2024). Of all the flaviviruses, dengue is of utmost concern worldwide owing to its vast spread to pandemic proportions (Abdalgader et al., 2024). DENV has four antigenically unique serotypes: DENV 1, DENV 2, DENV 3, and DENV 4, prevalent only in Southeast Asia (Abdullah et al., 2024). The epidemiology of the serotypes has spread, with the subtropical and tropical sectors of the world facing the combined effect of all four serotypes (Abbasi et al., 2024). The disease manifestation spectrum of dengue is wide, leading to the split of three stages of dengue: dengue fever (DF), dengue haemorrhagic fever (DHF) (severe dengue), and dengue shock syndrome (DSS) (severe dengue) (Alghsham et al., 2023; Abbasi et al., 2024).

In peripheral circulation, neutrophils are the most prevalent type of leukocyte (Acosta-Perez et al., 2022; Acchioni et al., 2024; Chua et al., 2024). The peripheral circulation of granular proteins and canonical proteins linked to neutrophil degranulation significantly increases in severe dengue patients (Zhang et al., 2023; Chua et al., 2024). One of the most notable clinical manifestations of dengue patients’ neutropenia may be a host defense mechanism. Neutrophil extracellular traps (NETs), a rare kind of neutrophil cell death, are present in robust levels in dengue patients (Chua et al., 2024; Meier et al., 2024). Neutrophil NET releases byproducts may aid in and enhance dengue pathogenesis (Chua et al., 2024; Meier et al., 2024). Neutrophils are activated during DENV infections; however, the function of neutrophils in the pathogenic etiology of dengue is still not fully known (Niranjan et al., 2019b; Meier et al., 2024).

Matrix metalloproteinases (MMPs), or matrixins of the metzincin protease superfamily, have great relevance as regulators of signalling networks in extracellular tissues (Niranjan et al., 2019b). An interplay amongst the three-organ system—the liver, vascular endothelium, and the immune system—serves as the key towards DHF and DSS pathogenesis (Niranjan et al., 2019a). Matrix metalloproteinases may be one of the inflammatory mediators that interact with the vascular endothelium. MMPs play a significant role in the beginning and development of dengue pathogenesis, but their precise functions are not entirely understood (Niranjan et al., 2019a; Chaiyana et al., 2020). DENV-infected immature DCs are known to enhance endothelial permeability by over generating MMP-2 and MMP-9 (Carmona-Rivera et al., 2015). Neutrophils are known to secrete immune modulators and their granule constituents, including NETs and different granules (MMP9, MMP2, NE, etc.). These immune modulators play a vital role in the initiation of apoptosis (Niranjan et al., 2019a).

DHF/DSS sometimes develops into acute respiratory distress syndrome (ARDS) in dengue endemic areas (Arankalle et al., 2024). Increased permeability of the alveolar-capillary barrier is a characteristic feature of ARDS (Bellissimo-Rodrigues and Dal Fabbro, 2023; Gimenez-Richarte et al., 2024). These epithelial cells at the alveolar lining serve as the first line of defense against toxins and infectious agents by regulating leukocyte influx (Dash et al., 2024). During initial inflammatory stages, the recruitment of activated neutrophils is considered detrimental, due to the stimulation of tissue injury by released proteases, oxidants, NETs along with other inflammatory mediators. Collateral pulmonary tissue damage in ARDS is thought to be triggered by excessive extracellular neutrophil granular enzyme production (Suchanti et al., 2023; Dash et al., 2024). The findings of Fligiel et al. (2006), showing elevated levels of MMP9, MMP2 & MMP8 in bronchoalveolar fluids (BAL), may indicate MMPs to be a contributor to or marker of Acute Lung Inflammation (ALI) (Dash et al., 2024). It was observed in patients with type 1 diabetes that metalloproteinase-2 and cardiovascular disease were independently associated with metalloproteinase-14, suggesting an important role of MMP-14 in other diseases (Melin et al., 2021). Another study has described the role of MMPs in apoptosis and its mechanistic involvement; however, the exact mechanisms of apoptosis associated with MMPs are still not clear and need extensive investigations (Caldeira et al., 2024).

The precise mechanism of neutrophil-mediated pathogenic etiology of severe dengue and acute respiratory distress syndrome (ARDS) is unknown (Sawant et al., 2023; Keshav et al., 2024). Matrix metalloproteinases are thought to play a significant role in both the progression of dengue and in ARDS (a key pathophysiological hallmark of severe dengue (Ahmad et al., 2023; Sawant et al., 2023)). Therefore, the present study focuses on identifying the exact cellular and molecular immune mechanisms of neutrophils, elucidating their pathogenic etiology in severe dengue and ARDS (Niranjan et al., 2024).

## 2 Materials and methods

### 2.1 Cell lines used in study

HL 60, a human peripheral blood pro-myeloblast cell line & A549, human lung epithelial cells, were acquired from the National Centre for Cell Sciences (NCCS), Pune, and nourished at the ICMR-VCRC, Tissue & Cell culture facility (Niranjan et al., 2019a).

### 2.2 *Culture and* maintenance of HL60 cell line

The human peripheral blood pro-myeloblast cell line, HL 60, was upheld in RPMI–1640 medium supplemented with 5%–10% FBS. The media were changed depending on the growth rate of the cells and the hardness of the media. The cell pellet was resuspended into a single-cell suspension by the addition of fresh medium. The cells were observed under phase-contrast inverted microscopy and placed in a CO_2_ incubator at 37°C with 5% CO_2_.

### 2.3 *Culture and* maintenance of the A549 cell line

Human lung epithelial cells, A549, were upheld in DMEM medium supplemented with 10% FBS. The media were changed depending on the growth rate of the cells and the hardness of the media. The cultured cells were washed with 1000 µl of fresh media without FBS. 3–4 ml of sterile medium supplemented with FBS was added to the T-25 flasks. The cells were observed under phase-contrast inverted microscopy and placed in a CO_2_ incubator at 37°C with 5% CO_2_.

### 2.4 MTT assay for cell viability

The MTT assay was performed to determine cell proliferation rate as per the protocol described by Morgan, 1998, with necessary modifications. The cell suspension was obtained using centrifugation and resuspended in fresh, prewarmed media at a minimum number of 1 × 10^6^ cells/ml. The cells were serially diluted in culture medium, and 100 µl of cells of each dilution were added to 96-well plates. After 24h of incubation, 10 µl of MTT was added to each well, including blanks. Following 3–4h incubation in a CO_2_ incubator at 37°C with 5% CO_2_, 100 µl of DMSO was added to the wells, and absorbance was measured at 570 nm.

### 2.5 Differentiation of HL60 cells into Neutrophils

A fresh passage of HL60 cells with a cell count between 7 × 10^5^ and 10 × 10^5^ cells/ml was plated onto a six-well microtitre plate. The cells were fed with 1.0% DMSO (dimethyl sulphoxide) and incubated in a CO_2_ incubator at 37°C with 5% CO_2_ for 2–3 days for neutrophil differentiation, with periodic examinations of the cells for differentiation under an inverted phase-contrast microscope^32^.

### 2.6 Exposure of HL60 with NS1 antigen of DENV type 2 and Atorvastatin

The NS1 antigen of DENV type 2 was procured from R & D systems and Atorvastatin was procured from Sigma. The recombinant antigen was reconstituted with sterile molecular-grade water to a final concentration of 1 µg/µl. Atorvastatin was reconstituted with sterile PBS to a concentration of 100 μM. The cells were treated with NS1 antigen per se to a final concentration of 10 µg/ml^33^ and NS1 (10 µg/ml) and 10 µM of Atorvastatin and Atorvastatin per se to a final concentration of 10 µM for a duration of 24h.

### 2.7 Exposure of A549 cells with secretome from HL60 cells exposed to various concentrations

The exposed HL 60 cells following incubation were centrifuged at 1200 rpm for 6 min at 4°C to separate the cell lysate and secretome (containing the expressed & released immune modulators and viral protein antigen). Five hundred microliter of supernatant/secretome were unveiled to the fresh passage of A549 cells on a six-well culture plate.

### 2.8 Cell toxicity assay

The MTT assay was performed to determine cell toxicity as per the protocol described by Marks et al., 1992, with necessary modifications. A549 single-cell suspension was obtained by centrifugation and resuspended in fresh, prewarmed media at a minimum of 6 × 10^3^ cells/ml. 100 μl of cell suspension was loaded onto the microtitre plate. Treatment of 100 µl of HL60 secretome was added to the wells. After 24h of incubation, 10 µl of MTT was added to each well, including blanks. Following 3–4h incubation in a CO_2_ incubator at 37°C with 5% CO_2_, 100 µl of DMSO was added to the wells, and absorbance was measured at 570 nm.

### 2.9 Exposure of A549 cells with recombinant MMP2 protein

Recombinant human MMP2 protein was procured from R & D Systems. The recombinant protein was reconstituted with nuclease-free water to a final concentration of 100 ng/μl. The cells were treated at varying concentrations of 1 ng, 10 ng and 100 ng for a duration of 24h.

### 2.10 Protein estimation

Protein estimation of secretome samples was executed using Bradford’s reagent. BSA (Bovine Serum Albumin) standard (2 mg/ml) was serially diluted in the ratio of 1:2. Estimation was done in duplicates. 5 µl of 1:5 secretome sample and standard were added to a microtitre plate and sterile molecular grade water was utilized as a blank. To this, 250 µl of Bradford’s reagent was added, placed on a rocker shaker for 30 s, followed by incubation at room temperature for 20 min, and OD was measured at 595 nm and net absorbance versus protein concentration was plotted. This standard curve graph was used to determine the concentration of protein in an unknown sample.

### 2.11 Immunoassay

Secretome levels of MMP2 were measured with the Human MMP-2 Duo-Set ELISA kit (R&D Systems) following the manufacturer’s instructions. Briefly, standard human MMP-2 and neutrophil secretome samples were incubated on a 96-well plate pre-coated with mouse antibody against human MMP-2 in duplicates. The MMP-2 was then probed by the addition of HRP-conjugated anti-human MMP-2 mouse antibody. After the addition of tetramethylbenzidine for 20 min, followed by stop solution, the OD was read at 450 nm using a microplate reader. To calculate the secretome MMP-2 concentration (ng/ml), the average reading between duplicate wells was subtracted by the blank.

### 2.12 RNA extraction and quantification

Cells were obtained through centrifugation and resuspended in trizol. RNA extraction was carried out through the TRIzol method with necessary modifications^36^. The RNA pellet was air-dried and reconstituted in 20 µl of sterile molecular-grade water. The RNA extract was quantified with the help of the Thermo Scientific µDrop™ Plate. 2 µl of sample is utilised for quantification and absorbance is measured at 230 nm. The RNA extract was stored at −20°C.

### 2.13 *Two-step* reverse transcriptase polymerase chain reaction and cDNA synthesis

The two-step RT-PCR was carried out for standardisation of annealing temperatures for all primers. The details of the primers are mentioned in **Table 1**. Preceding reaction set up, the annealing temperatures of all primers were calculated and expected target gene length was found using BLAST analysis. 300ng/µl of extracted RNA was utilized for cDNA synthesis (as per the protocol described by Applied Biosystems). The synthesised cDNA is used as template for conventional PCR. For each reaction 10 μl of master mix (Promega), template cDNA and 3 μl of molecular grade water were utilised. Primer concentration was 10 pmol. The polymerase chain reaction was set for initial denaturation at 95°C for 5 min, followed by 35 cycles involving denaturation at 94°C for 30 s, annealing at varying temperatures depending on primers for 60 s, extension at 72°C for 20 s, and a final extension step at 72°C for 7 min. The PCR products were subjected to agarose gel electrophoresis for DNA fragment separation. 2% agarose gel was utilized in this study. The identification of target gene amplification was done with the help of a molecular weight marker.

**Table 1:** Details of primers.

Oligo name	Sequence 5′–3′	T_m_ (°C)	Expected amplicon
Caspase 3 Forward	GAG CTG CCT GTA ACT TG	52	214 bp
Caspase 3 Reverse	ACC TTT AGA ACA TTT CCA CT	52	
Caspase 6 Forward	ACT GGC TTG TTC AAA GG	50	183 bp
Caspase 6 Reverse	CAG CGT GTA AAC GGA G	51	
BCL 2 Forward	AGG AAG TGA ACA TTT CGG TGA C	60	146 bp
BCL 2 Reverse	GCT CAG TTC CAG GAC CAG GC	65	
BAX Forward	TGC TTC AGG GTT TCA TCC AG	58	170 bp
BAX Reverse	GGC GGC AAT CAT CCT CTG	58	
BAD Forward	GAG TGA GCA GGA AGA CTC CAG C	66	341 bp
BAD Reverse	TCC ACA AAC TCG TCA CTC ATC C	62	

### 2.14 Real-time polymerase chain reaction

Real-time PCR (qRT-PCR) was performed for the relative expression analysis of various genes in response to DENV serotype 2 NS1 antigen-activated neutrophil stimulation. The synthesized cDNA is the template utilized for the reaction. SYBR Green is the dye exploited for nucleic acid staining. It binds to the minor groove of dsDNA and emits fluorescence. The fluorescence emitted in each cycle is proportional to the amount of amplicon present and therefore can be quantified. Five microliter of template was added to the reaction mixture containing SYBR Green master mix (Roche), Molecular grade water and primer. Primer concentration was 10 pmol. The samples were run in technical duplicates. The cycle threshold value (C_T_) of the gene of interest was normalized using the housekeeping gene GAPDH or β-actin. In this study, the β-actin gene was used as a reference for gene expression profile quantification and analysis.

### 2.15 Experiment with mice

BALB/c mice were treated with saline or recombinant NS1 of serotype-2 (50 μg/mouse) antigen alone or in combination with atorvastatin (40 mg/kg). Blood samples were collected and kept for RNA isolation. RNA isolation was done, and assessment of matrix metalloproteases and other genes was done by using real-time PCR. Gene expression was normalized with GAPD internal control. Tissue sections were collected, and immunocytochemistry of CD1 and other molecules (VE cadherin, a-SMA) was performed as per the protocol described earlier with our lab with desired modifications (Niranjan et al., 2022a). Trichrome blue stating was done for the collagen in the mice lung sections following the protocol as described by us previously (Upparahalli Venkateshaiah et al., 2019).

### 2.16 Statistical analysis

The data is analyzed and represented as mean ± SEM. The software GraphPad prism 5 was selected for the analysis needed for statistical purposes. The ANOVA test (one-way analysis of variance) was done, using Tukey’s test or the Newman-Keuls test as a *post-hoc* test. A < 0.05 *p*-value was accepted as statistically significant.

## 3 Results

### 3.1 Effect of NS1 antigen of DENV-2 on the protein expression in neutrophils and MMP-2 release

**Figure 1:**
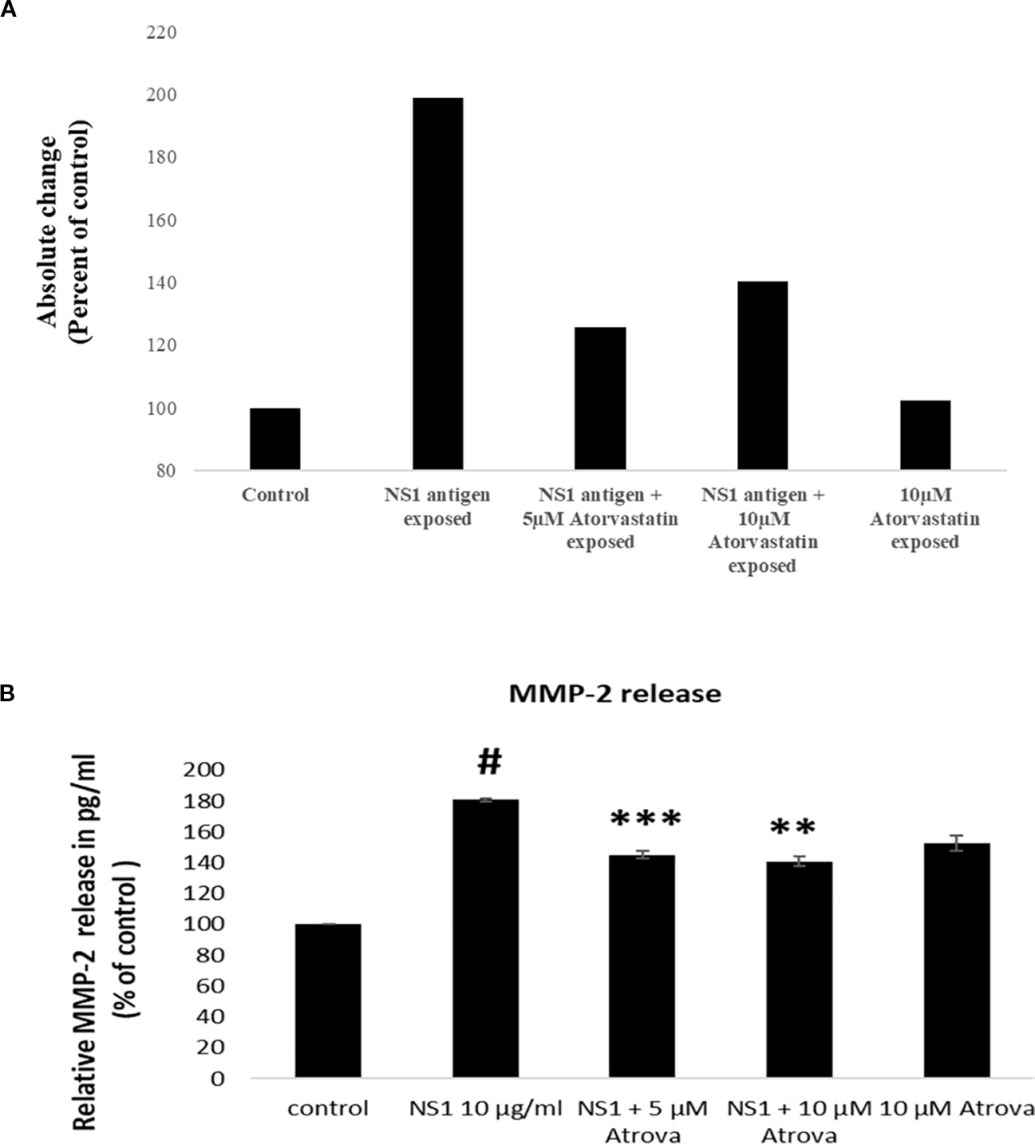
**(A)** Protein estimation of neutrophil cell secretome. Protein estimation of cell secretome obtained from HL60 cells, differentiated into neutrophils was carried out using ‘Bradford reagent’ as per protocol described by the manufacturer. Total protein content in secretome of HL60 cells differentiated neutrophil cell control and cells exposed to NS1 antigen and varying concentrations of atorvastatin. **(B)** MMP-2 estimation by ELISA in neutrophils’ secretome (culture medium harvested from NS1-treated neutrophils).

The protein content released by the HL60 cells, differentiated into neutrophils, was measured, combined with cell morphology assessment, upon exposure to NS1 antigen alone and in combination with atorvastatin. Protein estimation using Bradford’s reagent was done with the secretome harvested following 24h of exposure. As seen in **Figure 1A**, DENV 2 NS1 antigen has effectively increased the total protein content in a 24h of duration. Our experiment reveals that the secretome of NS1 antigen-treated neutrophil cells expresses higher total protein content (about 199%) than the control cells. We also found that higher concentrations of atorvastatin (10 µM) effectively decreased the total protein content in NS1 antigen-exposed neutrophil cells by about 140.4% when compared to the lower concentrations (5 µM).

Further to rule out whether neutrophils release MMP-2 protein in medium in response to dengue virus or not, the level of MMP-2 protein was measured in the secretome of NS1-treated neutrophils using the ELISA technique. As seen in the result (**Figure 1B**), we found that the MMP-2 level was markedly increased in the secretome of neutrophils subjected to NS1 antigen as compared with the control cells (non-stimulated cells). It was important to note that atorvastatin concentrations (5 and 10 µM) has significantly decreased the NS1-induced MMP-2 release by neutrophils in dose-dependent manner.

### 3.2 Effect of NS-induced neutrophil secretome on the apoptotic cell death of A549 lung epithelial cells

**Figure 2:**
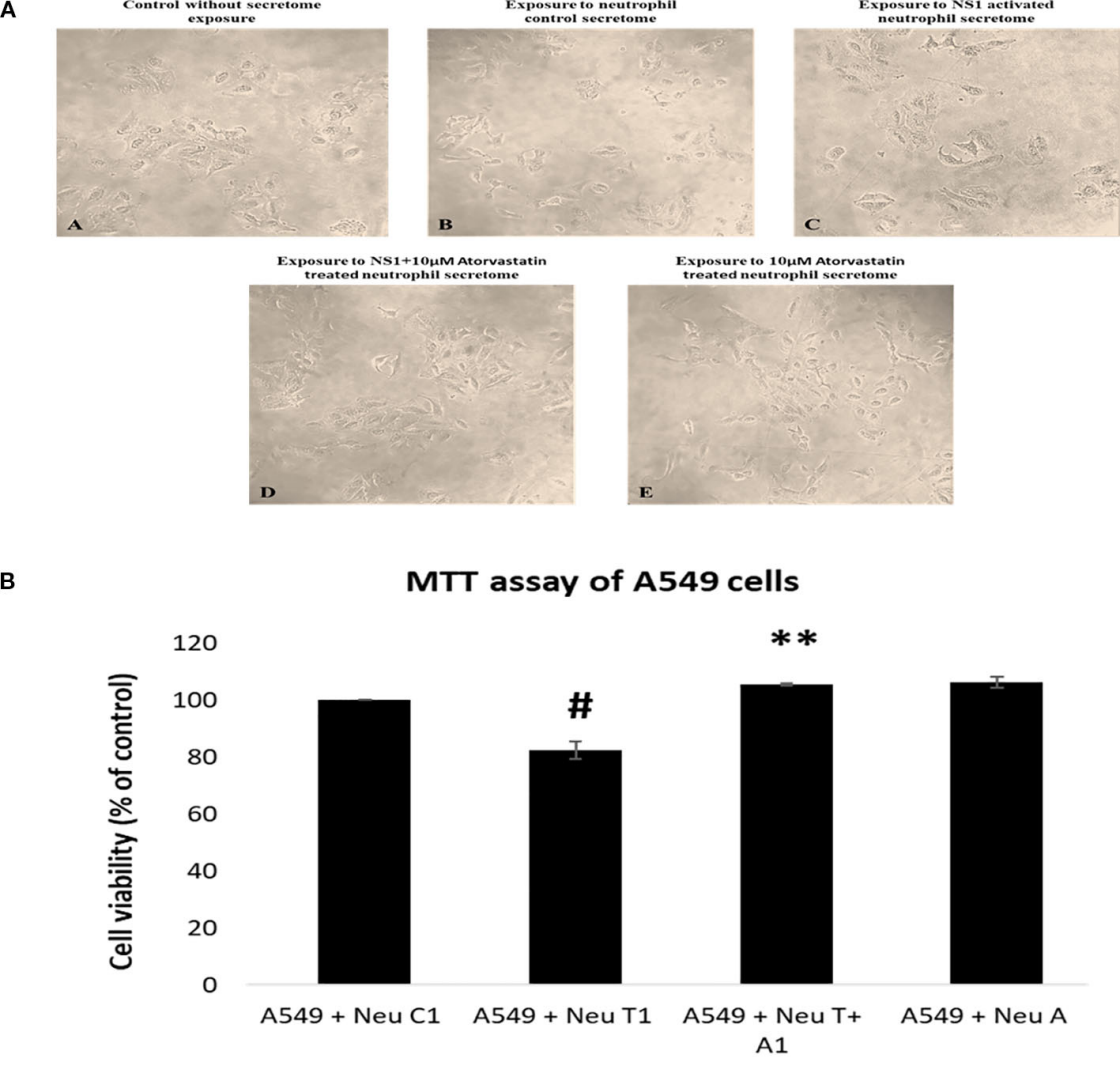
**(A)** Morphological assessment of A549 cells after 24h of exposure with neutrophil’s secretome. The morphology of epithelial cells upon exposure to neutrophil secretome was changed after a 24h time interval and observed at 20× magnification. Some epithelial cells upon exposure to NS1 exposed neutrophil secretome were found to be detached from surface of culture vessel and some were found to have changed morphology. (A) Control non-exposed cells, (B) cells after exposure to neutrophil control cell secretome, (C) cells after exposure to NS1 treated neutrophil secretome, (D) cells after exposure to secretome of neutrophil cells treated with NS1 + 10 µM atorvastatin and (E) cells after exposure to secretome of neutrophils cells treated with 10 µM of atorvastatin **(B)** Cell toxicity assay for A549 epithelial cells upon exposure to neutrophil secretome. Histogram represents the cell viability of A549 epithelial cells upon exposure to secretome harvested from treated neutrophil cells. #*p* < 0.05 compared with control and **p < 0.01 compared with NS1-treated group.

The various aspects of dengue pathogenesis remain unexplored, particularly of the lung pathophysiology and its immune cell-mediated mechanism of action. Immune cells are speculated to interact with other cells of the body and mediate the pathogenesis of dengue viral disease. It is well established that immune cells interact with other cells by releasing various cellular and molecular mediators. We have exposed A549 epithelial cells with the secretome harvested from NS1-induced neutrophil cells to understand the neutrophils-mediated cell death of lung epithelial cells. To assess this, the exposed cells were observed under the phase–contrast inverted microscope. As seen in **Figure 2A**, the secretome of NS1 antigen-treated neutrophil cells has caused A549 epithelial cell death. The A549 cells that were initially found attached to the culture surface were now detached and rounded up. Whereas, secretome of atorvastatin-treated neutrophil cells caused drastically reduced detachment of A549 epithelial cells from the culture surface. The same was concurred by performing cell toxicity MTT assay represented in **Figure 2B**.

### 3.3 Secretome obtained from NS1-stimulated neutrophils increased the mRNA expression pattern of apoptotic genes and total protein in A549 epithelial cells

**Figure 3:**
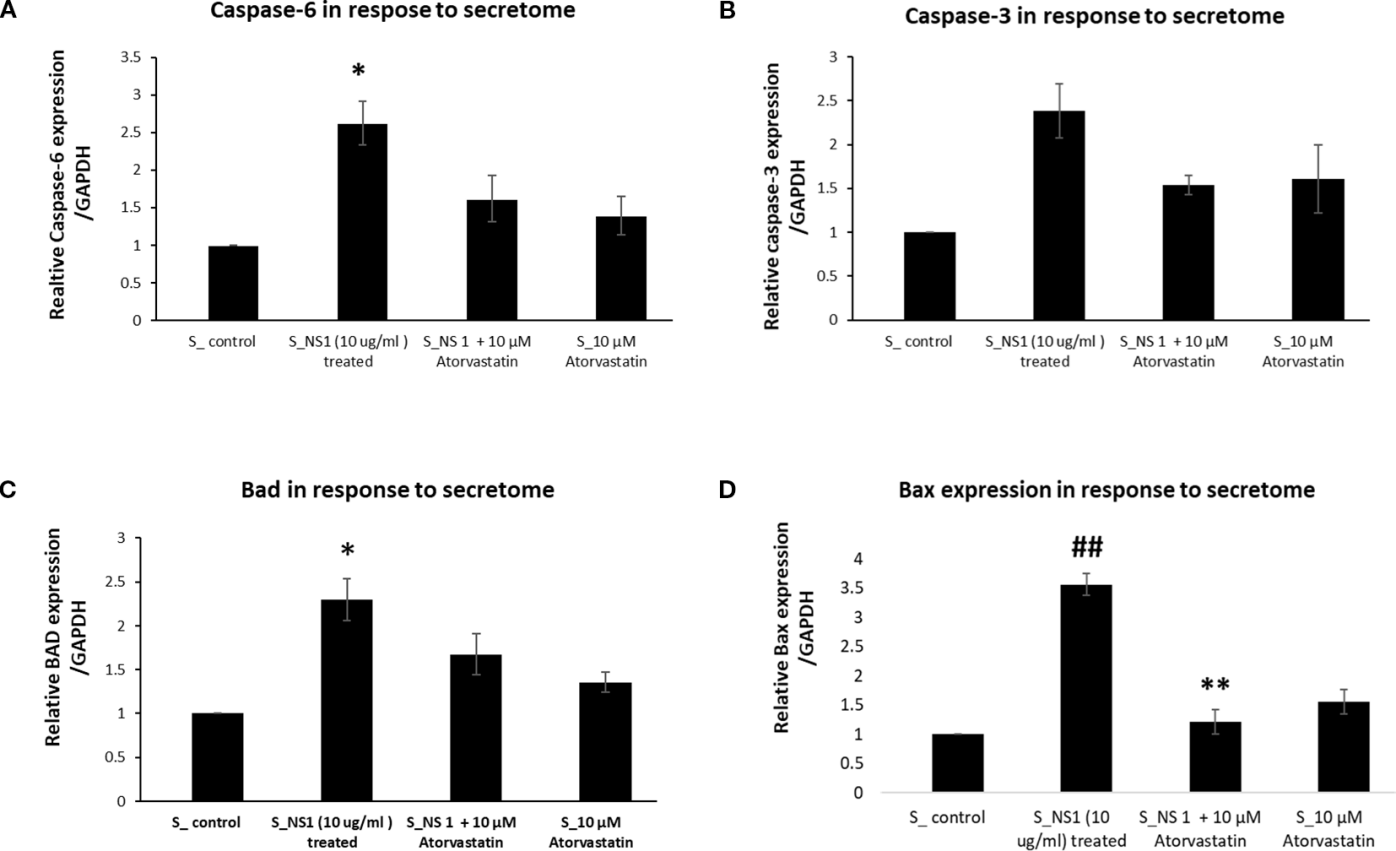
Expression profile of apoptotic genes exposed with secretome obtained from NS1 activated neutrophils. **(A)** Histogram represents the fold change of relative caspase-6 expression profile in A549 cells exposed with secretome obtained from neutrophil cells, after the treatment with NS1 and atorvastatin. *p < 0.05 compared with control **(B)** Histogram represents the fold change of relative caspase-3 expression profile in A549 cells exposed with secretome obtained from neutrophil cells, after the treatment with NS1 and atorvastatin. **(C)** Histogram represents the fold change of relative Bad-3 expression profile in A549 cells exposed with secretome obtained from neutrophil cells, after the treatment with NS1 and atorvastatin. *p < 0.05 compared with control. **(D)** Histogram represents the fold change of relative Box gene expression profile in A549 cells exposed with secretome obtained from neutrophil cells, after the treatment with NS1 and atorvastatin. ##*p* < 0.01 compared with control and **p < 0.01 compared with NS1-treated group.

Statins are found to exhibit inhibitory effects on DENV2-infected cells but their effect on the expression profile of various genes is not properly known. Therefore, we measured the expression profile of, Bax, and Cas6 genes in response to the secretome of neutrophils treated with Atorvastatin (10 µM) following NS1 antigen stimulation in lung epithelial cells after 24h of exposure. The expression of a reference gene or house-keeping gene was also checked to support the data. We found that, NS1 (10 µg/ml) stimulated neutrophil-secretome exposure increased the expression profiles of Bax, and Cas6 genes. The mRNA expression of Bax gene was reversed by atorvastatin (**Figure 3A**). In contrast, the mRNA expression of Cas6 genes remained unscathed (**Figure 3A**). Similarly, the caspase-3 expression was also reversed by the atorvastatin in a dose-dependent manner (**Figure 3B**).

Bad is involved in the activation of Bax, pre-apoptotic pore formers which are responsible for the mitochondrial outer membrane permeabilization (MOMP). Their activation leads to pro-apoptotic protein restrains. Therefore, we assessed Bad expression profile in NS1 (10 µg/ml) stimulated neutrophil secretome exposed epithelial cells following a 24h time interval. To support the data, the expression of a reference gene or house-keeping gene was also checked. We found that (**Figure 3C**), the expression profile of Bad exhibited an approximately threefold increase in cells interacting with NS1-stimulated neutrophil secretome compared to Bcl-2 exhibiting a minor increase in their gene expression.

Similarly, in addition to Bad expression profile of Bax gene was also measured. Their activation leads to pro-apoptotic protein restrains. Therefore, we assessed Bad expression profile in NS1 (10 µg/ml) stimulated neutrophil secretome exposed epithelial cells following a 24h time. To support the data, the expression of a reference gene or house-keeping gene was also checked. We found that (**Figure 3D**), the expression profile of Bax exhibited a ~3.5-fold increase in cells interacting with NS1 stimulated neutrophil secretome compared to Bcl-2 exhibiting a minor increase in their gene expression.

The protein content released by A549 epithelial cells upon exposure to neutrophil secretome was tested along with cell morphology assessment. Protein estimation was done with secretome harvested from A549 epithelial cell following 24h time interval upon exposure to neutrophil secretome. As seen in **Figure 3**, DENV-2 NS1 antigen exposed neutrophil secretome has effectively increased the total protein content of A549 epithelial cells in a 24h time interval. Our experiments disclose that treatment of epithelial cells with NS1 antigen exposed neutrophil secretome effectively increased the total protein content in epithelial cell secretome about (196%) and the exposure to secretome of atorvastatin (10 µM) treated neutrophil cells following NS1 antigen exposure effectively decreased the total protein content in epithelial cell secretome to about (161.4%).

### 3.4 MMP-2 exposure increased mRNA expression pattern of proapoptotic and apoptotic genes in A549, epithelial cells

**Figure 4:**
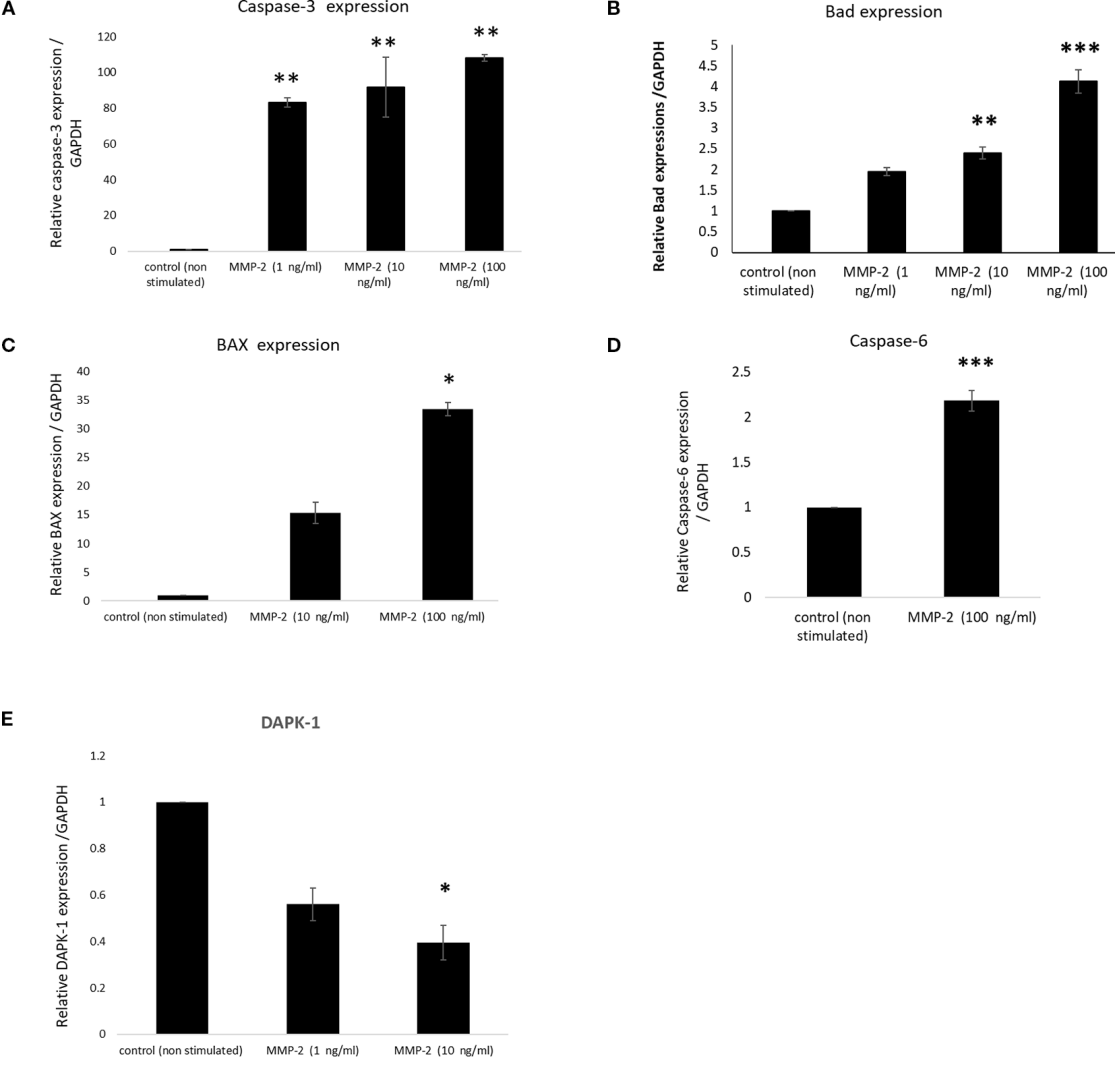
Expression profile of apoptotic genes in epithelial cells treated with varying concentrations of MMP-2. Histogram represents the fold change of gene upon normalization with GAPDH. Histograms represents the fold change genes upon normalization with GAPDH. *p < 0.05, **p < 0.01, ***p < 0.001, compared with control. **(A)** Histogram represents the fold change of relative Caspase-3 expression in A549 cells stimulated with different concentrations of MMP-2 (1, 10, and 100 ng/ml), compared with the non-stimulated control. p < 0.01 compared with control. **(B)** Histogram represents the fold change of relative Bad expression in A549 cells treated with MMP-2 (1, 10, and 100 ng/ml), compared with the control. **p < 0.01 and p < 0.001 compared with control. **(C)** Histogram represents the fold change of relative BAX expression in A549 cells treated with MMP-2 (10 and 100 ng/ml), compared with control. p < 0.05 compared with control. **(D)** Histogram represents the fold change of relative Caspase-6 expression in A549 cells exposed to MMP-2 (100 ng/ml), compared with the non-stimulated control. p < 0.001 compared with control. **(E)** Histogram represents the fold change of relative DAPK-1 expression in A549 cells treated with MMP-2 (1 and 10 ng/ml), compared with the non-stimulated control. p < 0.05 compared with control.

To know the effect of MMP2 in causing the cell death of lung epithelial cells we have tested effect of purified MMP-2 protein on the apoptosis of lung epithelial cells A549. We assessed the expression profile of caspase-3 and Bad in lung epithelial cells upon treatment with two different concentrations of MMP-2 (1, 10, and 100 ng/ml). we observed that caspase-3 expression was significantly high around 100-fold compared to control. However, MMP-2 concentrations of 1 and 10 ng/ml have significantly upregulated the caspase-3 gene expression to 80- and 90-fold increase, respectively. We found that (**Figure 4**) the expression profile of Bad was upregulated in cells treated with MMP-2 compared to control cells. Further cells treated with higher concentrations of MMP-2 (100 ng) observed a fourfold increase in Bad gene than the cells treated with lower concentrations of MMP-2 (1 ng). We assessed the expression profile of Bcl-2 in lung epithelial cells upon treatment with two different concentrations of MMP-2 (1 ng and 100 ng). The expression profile of Bcl-2 (**Figure 4**) was down regulated in cells treated with MMP-2 compared to control cells. Further cells treated with higher concentrations of MMP-2 (100 ng) exhibited a ~twofold decrease in the Bcl-2 gene than the cells treated to lower concentrations of MMP-2 (1 ng). Further Bax expression was also increased by nearly around 30-fold compared to the control. However, caspase-6 expression was increased to twofold to 2.25-fold compared to the control. It was interesting to note that the DAPK-1 expression has significantly downregulated compared to control.

### 3.5 Assessment of apoptotic effect of MMP-14, NS1 alone and in combination with Atorvastatin on A549 epithelial cells

**Figure 5:**
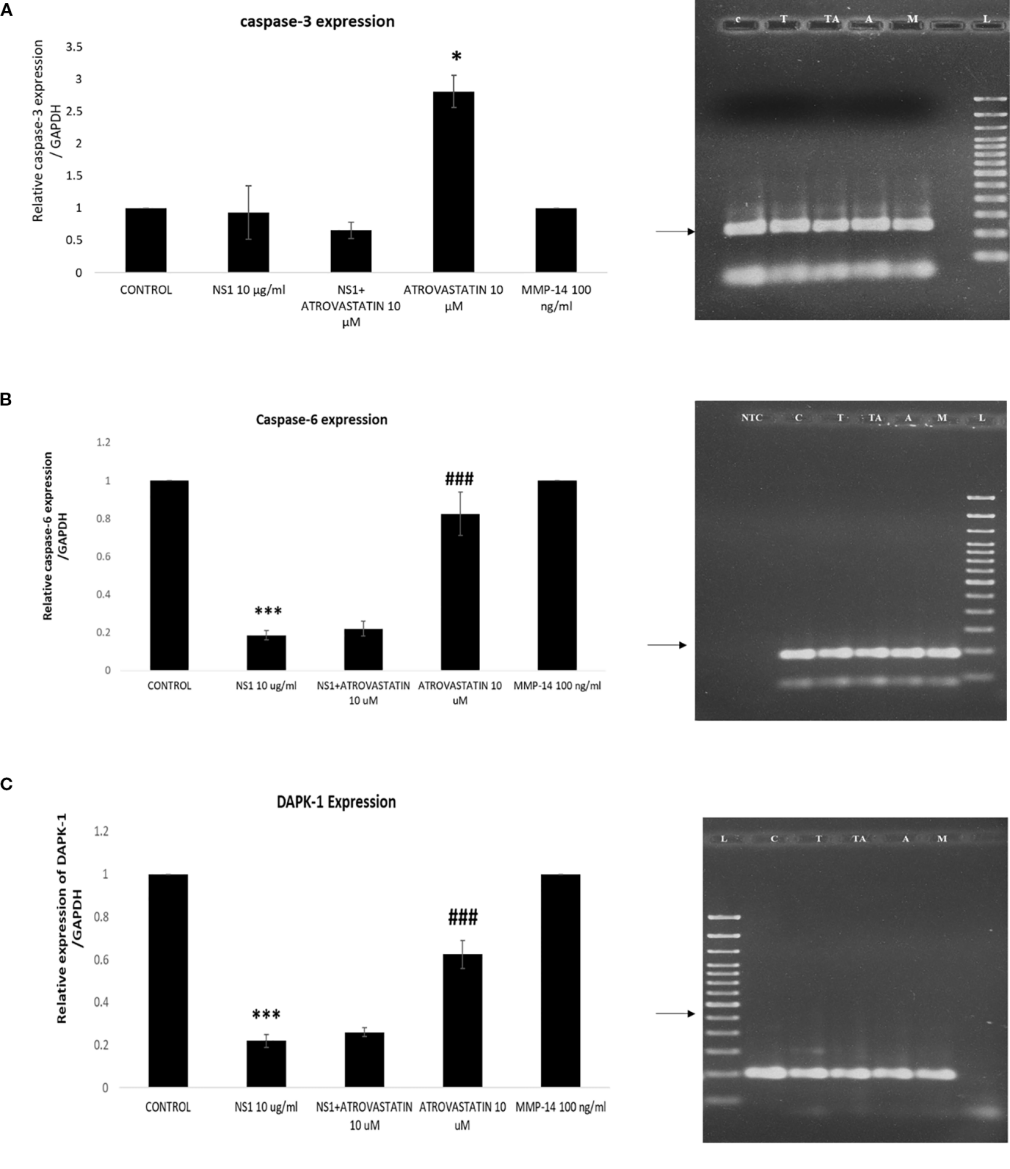
Expression profile of apoptotic genes exposed with NS1 alone and in combination with atorvastatin. **(A)** Histogram represents the fold change of relative caspase-3 expression profile in A549 cells exposed after the treatment with NS1 and atorvastatin. *p < 0.05 compared with control **(B)** Histogram represents the fold change of relative caspase-6 expression profile in A549 cells exposed after the treatment with NS1 and atorvastatin. ***p < 0.001, compared with control and ###*p* < 0.001, compared with control. **(C)** Histogram represents the fold change of relative DAPK-1 expression profile in A549 cells exposed after the treatment with NS1 and atorvastatin. ***p < 0.001, compared with control and ###*p* < 0.001, compared with control.

**Figure 6:**
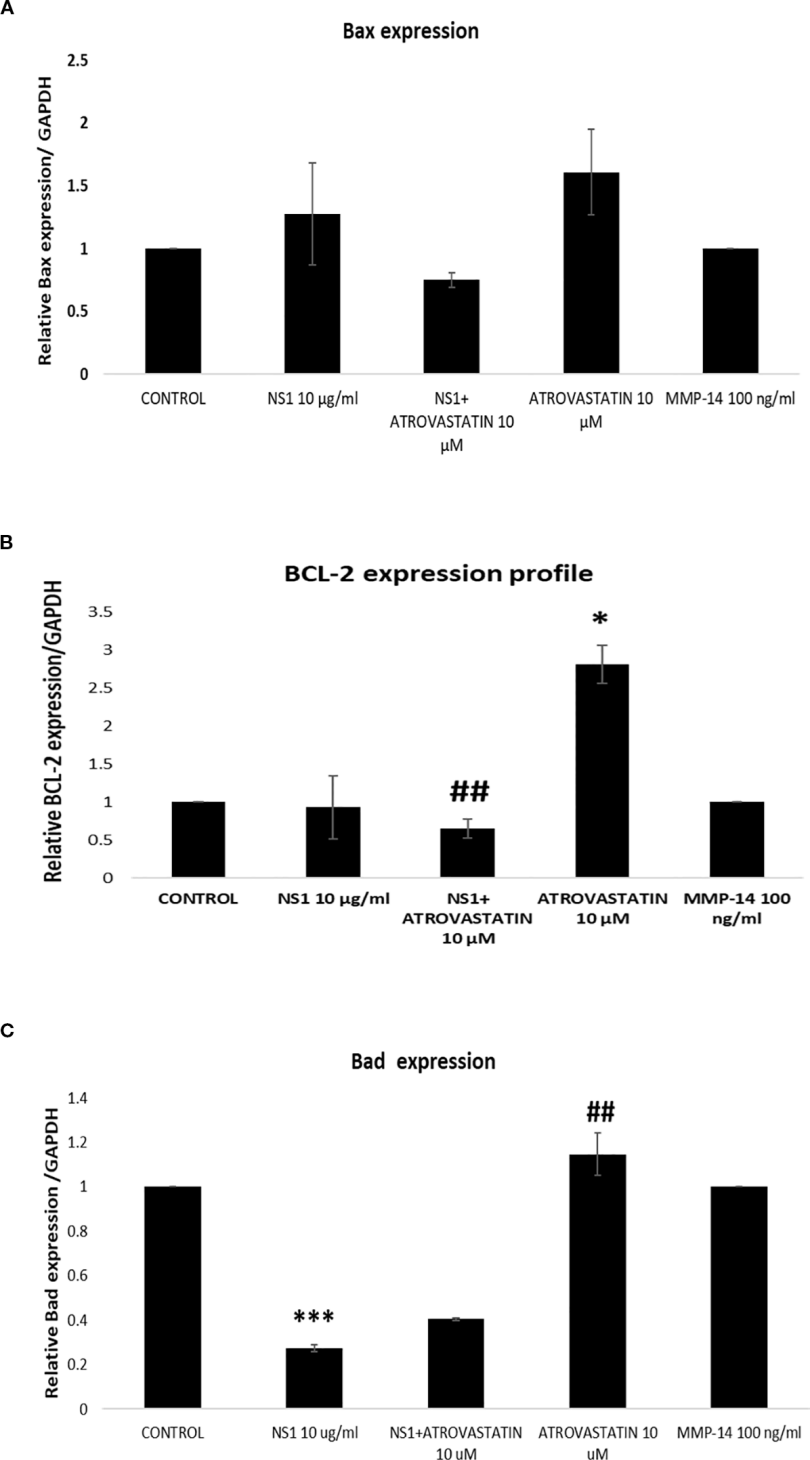
Expression profile of apoptotic genes exposed with NS1 alone and in combination with atorvastatin. **(A)** Histogram represents the fold change of relative Bax expression profile in A549 cells exposed after the treatment with NS1 and atorvastatin. **(B)** Histogram represents the fold change of relative BCL-2 expression profile in A549 cells exposed after the treatment with NS1 and atorvastatin. *p < 0.05, compared with control and ##*p* < 0.001, compared with NS1-treated group. ***P<0.001.

Further to know whether there is direct effect of MMP-14, NS1 on the apoptosis of A549 cells, exposure of MMP-14, NS1 alone and in combination with atorvastatin was done on A549 cells for 24h of duration. The mRNA expression profile of apoptotic genes was done in A549 cells. As seen in the **Figure 5A**, NS1 exposure downregulated the caspase-3 expression compared to control cells. Atorvastatin further in combination with NS1, further downregulated the expression profile of casepase-3. Similarly, NS1 exposure significantly caused downregulation of caspage-6 expression in A549 cells as seen in **Figure 5B**. Interestingly, as seen in the **Figure 5** MMP (100 ng/ml) does not cause any change in the apoptotic gene expression profile as compared to the control. In a similar fashion, the death associated proteinkinase-1 (DAPK-1) was also significantly downregulated in response to NS1 antigen as seen in the **Figure 5C**. These suggest that the NS1 antigen of dengue does not cause any apoptosis of lung epithelial cells. I addition to these genes the bas expression was also upregulated very little, and Bcl2 expression was decreased in response to NS1 antigen on lung epithelial cells however, the MMP14 does not cause any change in these genes’ expression profiles (**Figures 5**, **6**).

### 3.6 NS1 injection to the mice significantly increase neutrophils in mice lings and MMP2 expressions in blood

**Figure 7:**
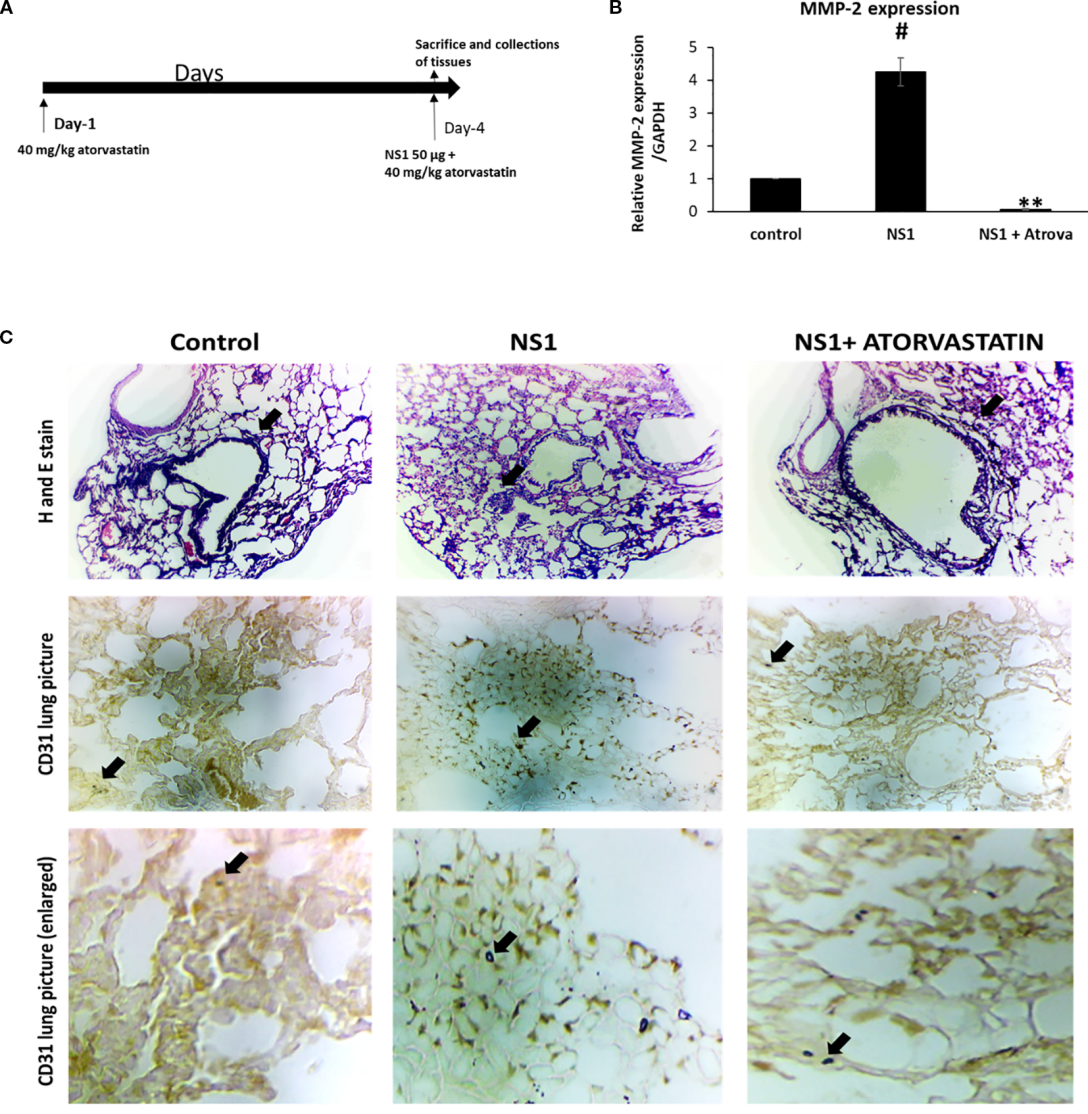
Experiment with Balb/c mice using NS1 alone and in combination with atorvastatin. **(A)** Diagrammatic representation of treatment plan for mice for NS1 antigen alone and in combination with atorvastatin. **(B)** Histogram represents the fold change of relative MMP-2 expression profile in mice blood exposed after the treatment with NS1 and atorvastatin. #*p* < 0.05, compared with control and **p < 0.001, compared with NS1-treated group. **(C)** Immunohistochemistry of the NS1-treated mice alone and in combination with atorvastatin. Upper panel shows the H&E staining and the lower panel shoes the CD31, also known as platelet endothelial cell adhesion molecule 1 (PECAM-1) expressed on leukocytes. The figure used here is one of the representative out of three independent experiments performed at least in duplicate.

To further proof the MMP-2 mediated mechanism of lung dysfunctions or ARDS in dengue pathogenesis we have tested this hypothesis in Balb/c mice using NS1 antigen alone and in combination with atorvastatin. As seen in the **Figure 7**, NS1 injection has significantly increased the expression of CD-31 positive cells (one of the marker of neutrophils) in mice lungs and the MMP-2 expressions in the blood. As seen in the figure atorvastatin has significantly caused the attenuation of CD31 positive cells as well as MMP-2 expressions in mice. CD31, also known as platelet endothelial cell adhesion molecule-1 (PECAM-1), is a protein moiety which helps remove the aged neutrophils from the one’s body. The CD31 is called as a cell adhesion molecule which is located on platelets, endothelial cells, and leukocytes. In the tissue sections images, it is seen that neutrophils are being interacted to the lung epithelial cells and thus may be releasing there MMP-2 molecules. These MMP2 molecules may further cause the apoptosis or distress to the lung epithelial cells in *in-vivo* situations. This proof complete mechanism of acute respiratory distress syndrome in severe dengue pathogenesis.

### 3.7 NS1 injection to the mice changes the VE cadherin, collagen, and a-smooth muscle actin (a-SMA) expression and localization in the mice lungs

**Figure 8:**
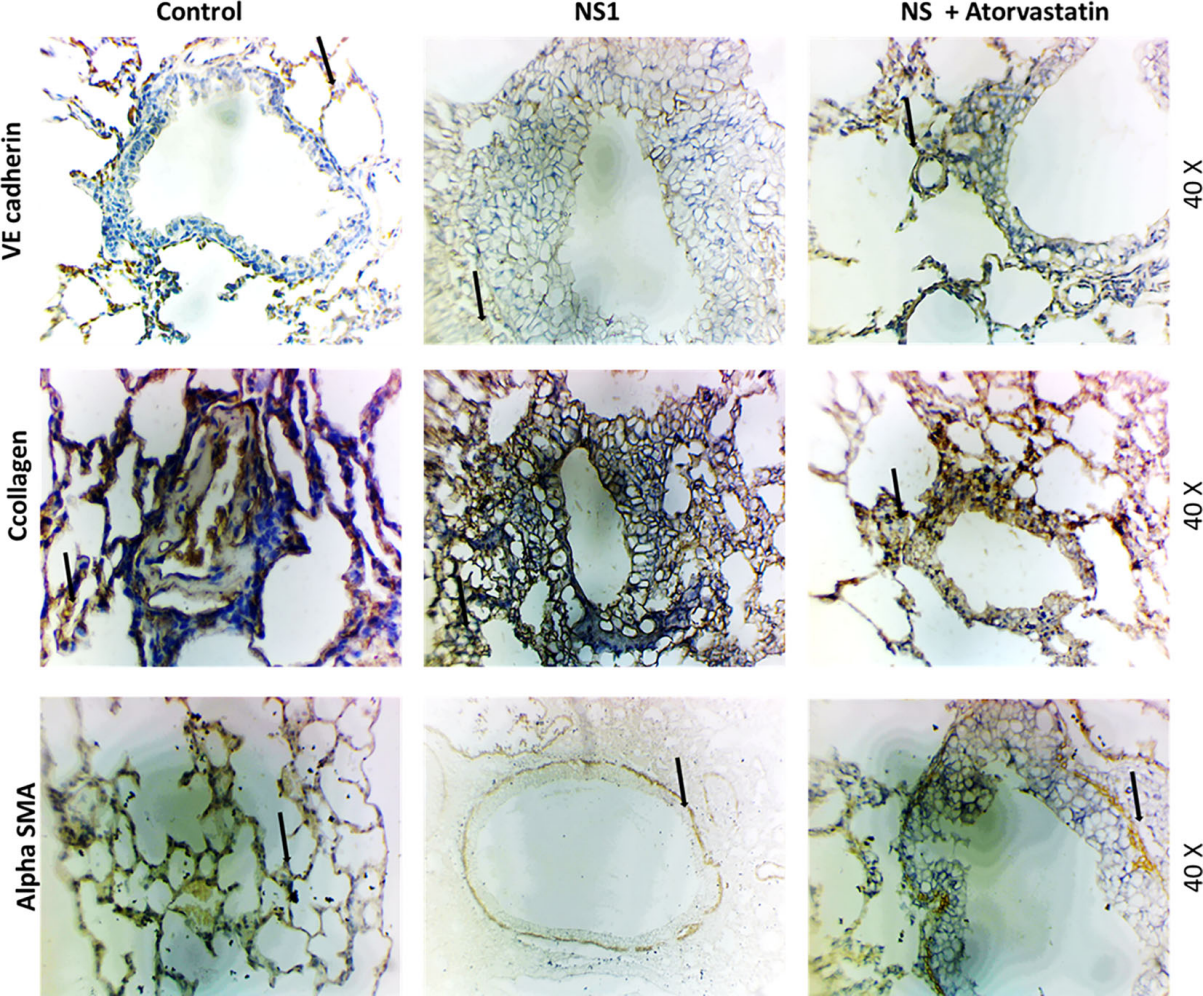
VE cadherin, collagen and a-SMA expressions and localisation in lungs of Balb/c mice using NS1 alone and in combination with atorvastatin. Immunohistochemistry of the NS1-treated mice alone and in combination with atorvastatin. Upper panel shows the VE cadherin staining, middle the trichrome blue stating for collagen and lower the a-SMA staining.

The lung skeleton is made up of VE cadherin, collagen and a-SMA and very important to maintain its proper physiological functions (Upparahalli Venkateshaiah et al., 2019). Any changes in its expressions and localization may result in the pathological outcome. To understand the structural homeostatic of the lung in dengue we have measured the VE cadherin, collagen and a-SMA protein expressions and localization using the immunocytochemistry and trichrome blue staining. The results can be seen in **Figure 8**. We have seen that VE-cadherin expression is increased in response to dengue antigen and atorvastatin significantly downregulated its expressions. a-SMA expression was also increased in the lungs of NS-1 injected mice lungs which was significantly downregulated in the presence of atorvastatin. Interestingly, it was observed that collagen staining was decreased in response to NS1 of dengue virus when compared with control. The collagen staining was remained unchanged in response to atorvastatin.

## 4 Discussion

In the present study, we show that the NS1 antigen of the DENV type 2 serotype significantly enhances the expression profile of toxic mediators in differentiated neutrophil cells (Rivera-Serrano et al., 2024). When these toxic mediators are exposed to the lung epithelial cells, they induce apoptosis of the lung epithelial cells, revealing the neutrophil-mediated effects of lung-associated pathology in severe dengue disease (Rob et al., 2024). Treatment with atorvastatin has significantly reversed the NS1 antigen-induced release of toxic mediators by neutrophils as well as further inhibition of apoptosis of the lung epithelial cells due to its toxic mediators (Kraus et al., 2024; Liu et al., 2024; Riwu et al., 2024).

It is well known and established that there is a distinct protein cascade involved extensively in the intrinsic apoptotic pathway involved in severe dengue pathogenesis; however, their cellular mechanisms are still poorly understood (Rocha et al., 2017; De Ramon et al., 2024; Melo et al., 2024). The process of apoptosis, with the Bcl-2 protein cascade as centre, has been vigorously understood and established that they induce Cyt C release by the mitochondria along with other factors (Chipuk and Green, 2008; De Ramon et al., 2024; Rodriguez et al., 2024). One of the many ways that severe dengue manifests itself is through ALI/ARDS characterized by epithelial cell distress (Al Matni et al., 2024; Kraus et al., 2024). Neutrophils are well known for their decisive role in viral dissemination, and concerning dengue, activated neutrophils are known to secrete matrix metalloproteases (Niranjan et al., 2022b; Kraus et al., 2024; Sul et al., 2024). In our previous study we have shown that, mRNA expression profile of matrix metalloproteases, including MMP2 are highly upregulated in neutrophils in response to dengue NS1 antigen (Niranjan et al., 2022b). However, what is the specific role of these matric metalloproteases was not known. In the present study we found that, MMP-2 is secreted by the neutrophils when they are activated by dengue NS1 antigen and it may cause sever dengue pathogenesis (Niranjan et al., 2019a; Niranjan et al., 2022b).

**Figure 9:**
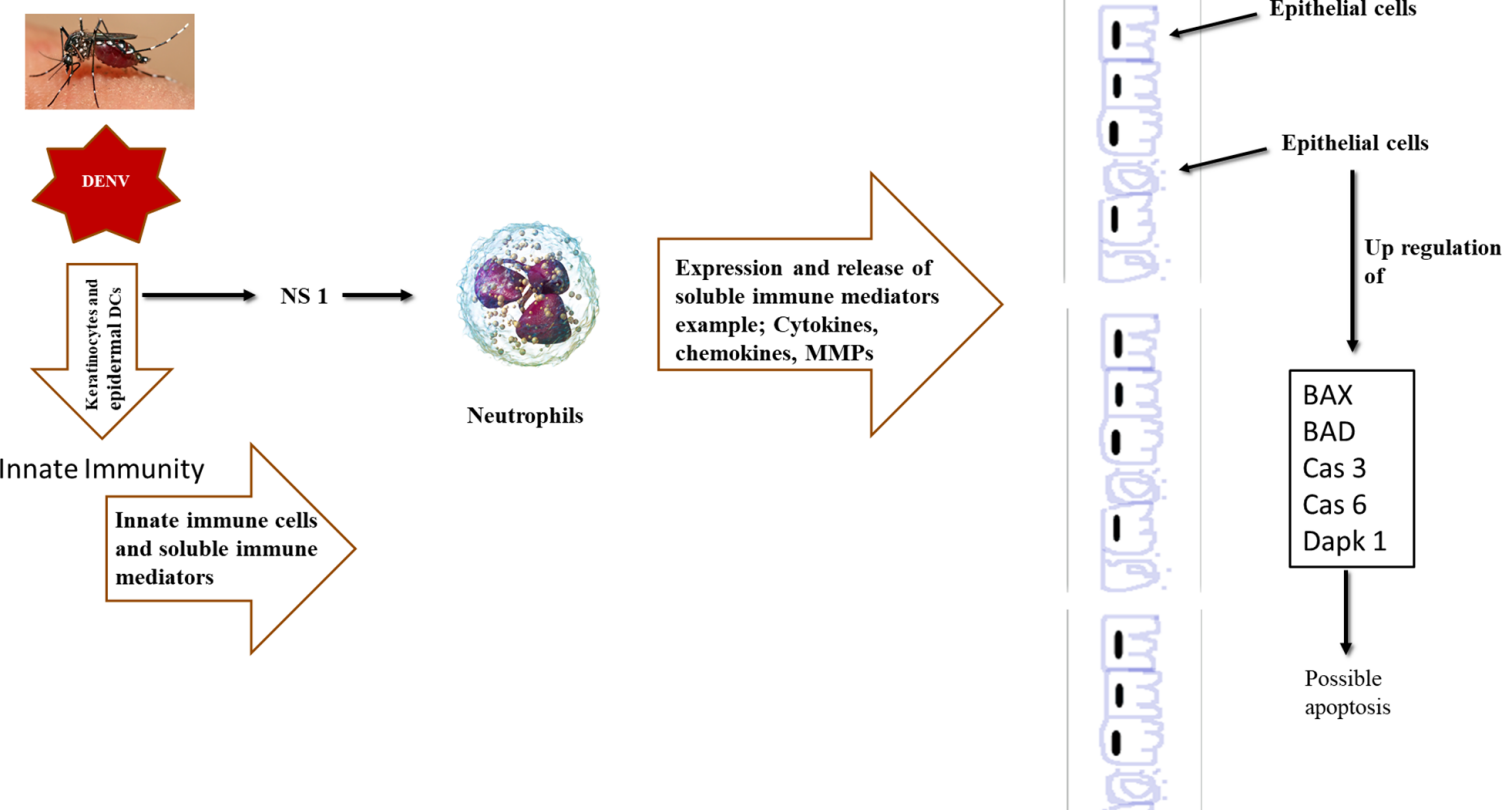
Diagrammatic representation of study outcome. As seen in the figure the dengue virus/antigen interact with the neutrophils and stimulate it for the release of matrix metalloproteinases. These matric metalloproteinases caused the cells distress or death of lung epithelial cells suggesting the mechanism of pathogenesis of severe dengue viral disease and acute respiratory syndrome (ARDS).

In the present study, neutrophil induced with NS1 antigen has secreted various toxic mediators in the secretome and it causes Bcl-2 gene alterations in epithelial cells leading to apoptosis or death (Melo et al., 2024). The critical step in a cell committing toward apoptosis following an intrinsic stimulus, is the oligomerization of the pro-apoptotic pore formers (Bax, Bak) leading to MOMP and the release of apoptotic factors (Wang et al., 2024; Abukhalil et al., 2025). This process is regulated by the BH3—only proteins (Bad, Puma, Noxa) by dislodging the pore-formers from anti-apoptotic proteins and allowing their oligomerization (Chauhan et al., 2018; Wu and Guo, 2024). This BH3-only proteins are classified as activators and sensitizers where the activator protein (Bid, Puma) binds to both pro-apoptotic and anti-apoptotic proteins which increases the possibility of apoptosis prevention and sensitizer proteins (Bad, Noxa, and Bmf) bind only to anti-apoptotic proteins and indirectly cause MOMP (Arnoult et al., 2005; Azad and Storey, 2013; Dadsena et al., 2021). In this study, secretome (collected from NS1-stimulated neutrophils) has significantly upregulated the expression of Bad and Bax expression in epithelial cells (**Figures 3C, D**) which indicates apoptosis initiation (Azad and Storey, 2013; Wang et al., 2024; Wu and Guo, 2024). Similarly, Caspases are aspartate-specific cysteine proteases that are primary mediators of apoptosis following extrinsic and intrinsic stimuli (Cao et al., 2022). Cas 3 and 6 are the effector caspases that coordinate the execution of apoptosis along with Cas7 (Hamacher-Brady and Brady, 2015). Their expression by cells would imply that the cell is in the execution stages of apoptosis. In this study, our results reveals that, there is a significant increase in the caspase-6 gene expression and in caspase-3 (**Figures 3A, B**) in epithelial cells exposed to secretome from NS1-activated neutrophils (Zhang et al., 2024). This confirms that the NS1-induced secretome of neutrophils caused cell distress or apoptosis of lung epithelial cells and may be one of the key cellular mechanisms of dengue pathogenesis in acute respiratory distress syndrome (ARDS in dengue). The Final study outcome is represented in **Figure 9**.

It was further important to know that, which individual molecule in neutrophils is responsible for causing apoptosis of lung epithelial cells, as there may be many toxic molecules present in this secretome (NS1-induced culture medium of the cells). As we have speculated that MMPs (especially MMP-2) may be responsible for causing the epithelial distress or epithelial apoptosis and may be responsible for the development of severe form of acute respiratory distress syndrome. Several studies have recently described the apoptotic causing property of MMP-2 in various disease setups (Das et al., 2024; Ibraheem, 2024; Kurbatova et al., 2024). In a study, it was seen that inhibition of MMP-2 attenuated ER stress-mediated apoptosis cell death describing the apoptotic property of MMP-2 (Bassiouni et al., 2025). Another study has described the apoptotic causing effects of MMP2 on DNA damage, of human lens epithelial cells, mediated by oxidative stress (Deng et al., 2024). Thus, to proof exact role of MMP-2, the apoptotic effects of purified MMP-2 was measured on A549 lung epithelial cells. We found that, MMP-2 concentrations have significantly upregulated the expression profile of apoptotic genes, that is, caspase-3, Bad, Bax, caspase-6, and DAPK-1 in lung epithelial cells suggesting its apoptosis causing property in dengue pathogenesis. This confirms that neutrophils mediated MMP-2 causes the lung epithelial cells death/distress and may be one of the important mechanisms of dengue pathogenesis associated to lung dysfunctions or ARDS. The present study is also supported by the existing literature and aligned with the apoptotic effects of MMP-2.

In deeper understanding of dengue pathogenesis, it becomes the obvious question whether NS1 antigen alone or other MMPs like MMP-14 might have some adverse effects on lung epithelial cells. Therefore, to proof the direct effects of NS1 alone and MMP14 was observed on lung epithelial cells in causing its apoptosis. As seen in the **Figures 5** and **6**, we found that NS1 alone done not cause the any increase in the expression profile of apoptotic gens suggesting NS1 alone or dengue virus alone does not cause any effects of the apoptosis of the lung epithelial cells. Furthermore, similarly MMP-14 has also caused very negligible effects on the apoptosis of lung epithelial cells. This suggested that that immune cells especially neutrophils activation is important in causing the sever lung distress or ARDS in dengue pathogenesis. There is similar study which also shoes that MMP-14 does not cause cell death but cause cell proliferation supporting our current findings (Liang et al., 2024; Lo et al., 2024; Wang et al., 2024). The protective role of statins has already defined in the literature suggestions their beneficial roles which also aligned with our current findings (Pinho-Ribeiro et al., 2017).

Many factors within our body contribute to viral replication cycle such as intercellular adhesion molecules, cholesterol, and isoprenylated proteins (Denoyelle et al., 2003; Djuric et al., 2010; Piani et al., 2023). Statins are known to be inhibitors of the synthesis pathway of these molecules and are also found to exhibit pleiotropic effects (Ferretti et al., 2017; Eliasson et al., 2019). This fact was exploited to understand the inhibitory effects of statins on viral replication. The work done by del Real et al., 2004 reiterates the decrease in disease severity upon statin usage in patients infected with HIV (Abdelghafar et al., 2022; Kalra et al., 2023). Martínez-Gutierrez et al., 2011 work suggests that statins may be involved in delaying assembly of DENV upon transcription and translation in *in-vitro* cultures (Allela et al., 2024). *In-vitro* data of the work done by Bryan-Marrugo and his colleagues on Huh 7 hepatoma cells infected with DENV 2 suggests that 48h after statin treatment to the DENV2 infected cells, various statin including, lovastatin, atorvastatin, Fluvastatin, and simvastatin exhibited inhibitory effects (Bryan-Marrugo et al., 2016; Ismail et al., 2024). In this study, atorvastatin (10 µM) has significantly shown its protective effects on the neutrophils mediated apoptosis of lung epithelial cells (Malhi et al., 2021; Nishimura et al., 2025).

It is further interesting to note that, the neutrophils are activated in the lungs and may cause acute respiratory distress syndrome (Chua et al., 2024). To proof this experimentally we have used mice and injected with dengue virus NS1 antigen alone and in combination with the atorvastatin. As seen in the **Figure 7** CD31 positive neutrophils come to the lungs and infiltrate to the lungs in different parts. This shows that neutrophils associated to the lungs interacts the lungs and causes various levels of MMP2 secretions (Liu and Rodgers, 2022). The secreted MMP-2 thus may cause the death or distress of lung epithelial cells causing the acute respiratory distress syndrome. This proofs that neutrophils mediated MMP2 release caused the ARDS in sever dengue pathogenesis. Tissue remodeling might have caused by the activated MMP-2 leading to lung physiological dysfunctions. Existing literature also strongly supports that neutrophils associated mediators are associated with the severity of disease (Chia et al., 2022). It was recently shown that, dengue virus infection in mice induces myeloid cell differentiation from bone marrow and generates Ly6Glow immature neutrophils with modulated functions (Duggal et al., 2024). This study strongly supports that there are infiltrations of some new neutrophils in the mice lungs in response to dengue virus/antigens and may cause acute respiratory distress symptoms. Structural protein expression (like collagen also a-SMA, and VE cadherin) in lungs are very important to maintain its physiological functions and any disturbances in these can results pathophysiological outcomes (Upparahalli Venkateshaiah et al., 2019). In the present study, we have seen that these is changes in these proteins expression in the lungs which might have caused a big pathological mechanism responsible for causing tissue remodeling leading to ARDS (Keshav et al., 2024; McBride et al., 2024).

In conclusion, it may be said that activated neutrophils are actively involved in the induction and execution of epithelial cell distress or apoptosis. MMP-2 and not MMP-14 causes mainly the apoptosis of lung epithelial cells. The exposure of epithelial cells to the secretome from NS1-activated neutrophils leads to the upregulation of Bax, Bad, Caspase genes in lung epithelial cells indicating distress or their cell death. This suggests that, the interaction of NS1 activated neutrophils with the alveolar epithelial cells may partially participate in the development of acute respiratory distress syndrome (ARDS) in dengue viral disease. The exposure of epithelial cells by atorvastatin significantly decreased the apoptotic mediators by neutrophils, including MMP-2 and protecting apoptosis of lung epithelial cells in response to dengue virus antigen. This suggests that atorvastatin may help in protecting the epithelial cell lining by inducing the overexpression of anti-apoptotic markers. The current findings are very noble, and we are reporting this mechanism for the first time. Our findings further highlight the MMP-2 as an important potential target for the development of dengue therapeutics. The present results are encouraging; however, further investigations may be required to clarify the findings.

## Data Availability

The raw data supporting the conclusions of this article will be made available by the authors, without undue reservation.
